# 

**DOI:** 10.1192/bjb.2024.11

**Published:** 2024-08

**Authors:** Stephen Potts

**Affiliations:** Consultant in liaison psychiatry in the Department of Psychological Medicine, Royal Infirmary of Edinburgh, Edinburgh, UK. Email: stephen.potts@nhslothian.scot.nhs.uk

**Figure:**
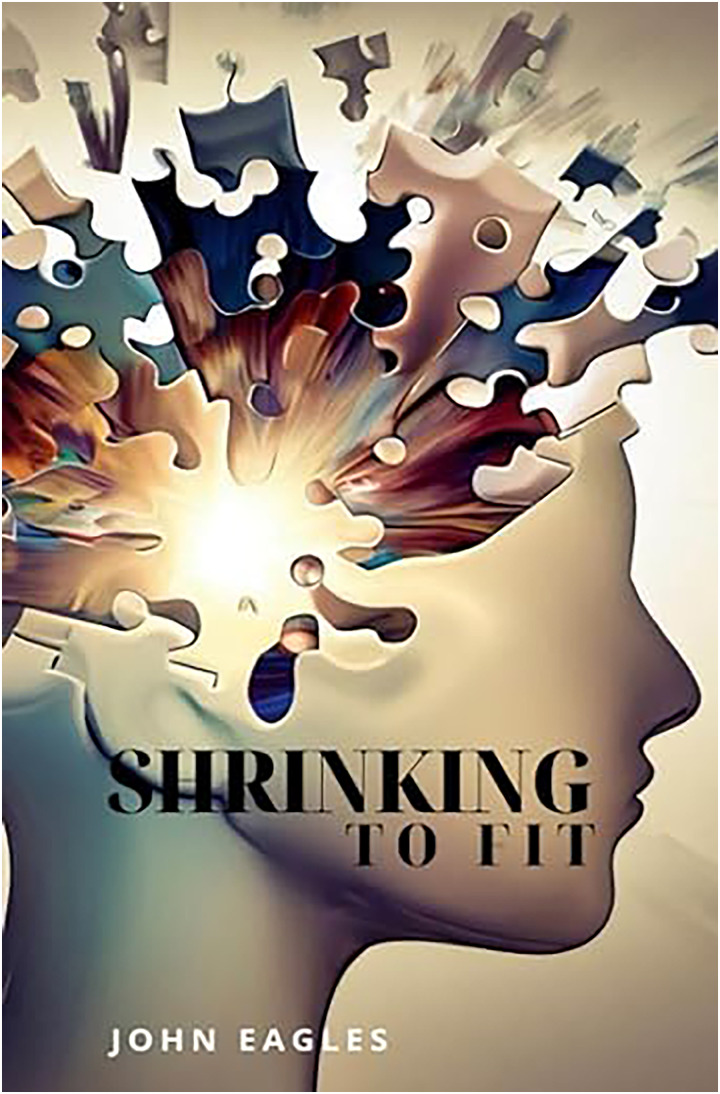

Write what you know, we are told. John Eagles, a retired NHS consultant psychiatrist and honorary professor in Aberdeen, has done just that. His second novel is set in an unnamed Scottish university department of psychiatry in the late 1980s. His protagonist, Douglas Barker, survived a scrape with the GMC in the prequel, and is now a new senior lecturer there.

*Declaration of interest 1:* Eagles was a long-standing big fish in those particular waters, where I – briefly – swam as a minnow. Our wakes did not cross, which is perhaps why I did not see (or failed to recognise) the darker parts of the pool that are essential to his plot. I caught glimpses at most, and not nearly Eagles’ full-blown monsters. The prioritisation of research above clinical work and teaching is a common allegation. Biased reviewing of each other's grant applications and papers, to see off competitors or promote allies, is an allegation less commonly made, because it requires an insider's perspective. And yes, out-and-out falsification of statistics occurs – but as a matter of course?

A new drug, strongly reminiscent of the SSRIs, is near to licensing via an unholy alliance of academic corruption and profit-driven Big Pharma, leaving Barker torn between his career and his principles. In the background are the mundane domestic pressures of wet weekends in a Fife caravan, low-key marital tensions and small-beer office politics. These were not enough to hold this reader until the corruption story got going halfway through, but then the hold was not strong. There are two antagonists, both Professors of Psychiatry. One is building a career scanning the brains of people with schizophrenia and the other is in hock to the drug makers. Both are corrupt. A third Professor reeks of stereotyping. Psychotherapist? Check. Beard? Check. Pipe? Check. He is even called Prof. Wiseman. (The bean-counter manager who marginalises him into retirement is a Mr Penman.)

But my attention wandered more because of the writing style than the content. Ian McEwan's novel *The Children Act* brilliantly describes major social issues through a judge torn between professional courses of action in the middle of a marital crisis. So it can be done. Doctors, especially psychiatrists, sometimes stand accused of assigning people to diagnostic boxes. Critics and reviewers face the same charge – of pigeonholing by genre. But there is merit in classification. McEwan's book justifies the label literary fiction, to which Eagles does not aspire. *Shrinking to Fit* has funny moments, but not enough to class as comedy. The action does not move fast or far, with stakes too low to count as a thriller. It is too realistic for satire. So what is it? I thought I heard axes being ground, and sensed a faint whiff of score settling, suggesting a *roman à clef.* It may be best to think of it as Medical Story NOS.

*Declaration of interest 2:* Medics are drawn to writing, during or after their medical careers. I have felt this draw, with modest results. Medical authors often write about their work, reflecting the interest of peers and public in their subject matter. Eagles’ peers, especially those of that time and place, may prize his story for the content. It stands as an example of what it is possible to do via creative writing courses and sustained effort.

